# Severe deforming dermatitis in a kitten caused by *Caryospora bigenetica*

**DOI:** 10.1186/s13028-021-00604-z

**Published:** 2021-10-10

**Authors:** Seppo Saari, Kirsti Schildt, Sanna Malkamäki, Ulla Andersin, Antti Sukura

**Affiliations:** 1 Veterinary Histopathology Laboratory Patovet, c/o Vita Laboratories, Laivakatu 5 F, 00150 Helsinki, Finland; 2 Evidensia Animal Hospital Tammisto, Tammiston Kauppatie 29, 01510 Vantaa, Finland; 3 Department of Veterinary Biosciences, University of Helsinki, P.O. Box 57, 00014 Helsinki, Finland; 4 Evidensia Veterinary Clinic Salovet, Horninkatu 7, 24100 Salo, Finland

**Keywords:** Apicomplexan, *Caryospora*, Cytology, Dermatitis, Feline, Histopathology, Protozoa

## Abstract

**Background:**

*Caryospora bigenetica* is an intracellular protozoan parasite, which in its primary hosts, typically snakes, is found it the intestine. Extraintestinal multiplication with the development of tissue cysts takes place in secondary hosts, which are normally prey for snakes. Natural infection in domestic animals has been reported only in dogs; this is the first report of *C. bigenetica* infection in a cat.

**Case presentation:**

A stray kitten developed nodular dermatitis after being adopted by a shelter. Firm swelling, nodules, and crusts were present mainly on the nasal bridge, eyelids, and pinnae. Histopathology and cytology revealed severe pyogranulomatous inflammation with abundant intracellular organisms suggestive of apicomplexan protozoa. Treatment with clindamycin 13 mg/kg twice daily was initiated, but the cat was euthanized because of the worsening condition. Transmission electron microscopy confirmed parasite’s apicomplexan origin postmortem, and the causative agent was identified as *C. bigenetica* by polymerase chain reaction and DNA sequencing.

**Conclusions:**

We present the first case of a naturally occurring infection with *C. bigenetica* in a cat. Although the definitive etiological diagnosis relied on molecular identification, the abundance of unsporulated oocysts and caryocysts and the parasite's effective reproduction within macrophages and in several other cell types might have enabled differentiation from other protozoal infections and allowed a presumptive diagnosis through cytology and histopathology.

## Background

Coccidian parasites of the genus *Caryospora* are intracellular intestinal parasites that infect reptiles and raptorial birds [1, 2]. Due to their low veterinary and economic impact and the host range that only rarely includes domestic animals, the genus has traditionally received less attention than many other coccidian parasites [1]. The genus includes *Caryospora bigenetica*, and this subspecies has various snake species as the primary host. Transmission of the parasite to snakes may occur through ingestion of oocytes after fecal excretion or through ingestion of an infected rodent. Rodents may become infected after ingestion of sporulated oocysts of fecal origin or by ingestion of an infected intermediate host (typically a rodent). In both situations, extraintestinal multiplication of the parasite occurs with the development of tissue cysts. Non-rodent species may accidentally become infected when ingesting either oocysts or tissue harboring parasitic cysts [1, 2]. Successful experimental transmission has been documented in mice [1, 3], cotton rats [1], swine [3], and dogs [1], while natural infection has only been described in dogs [4, 5]. The present case is the first report of natural infection in a cat to the best of our knowledge.

## Case presentation

**Fig. 1:**
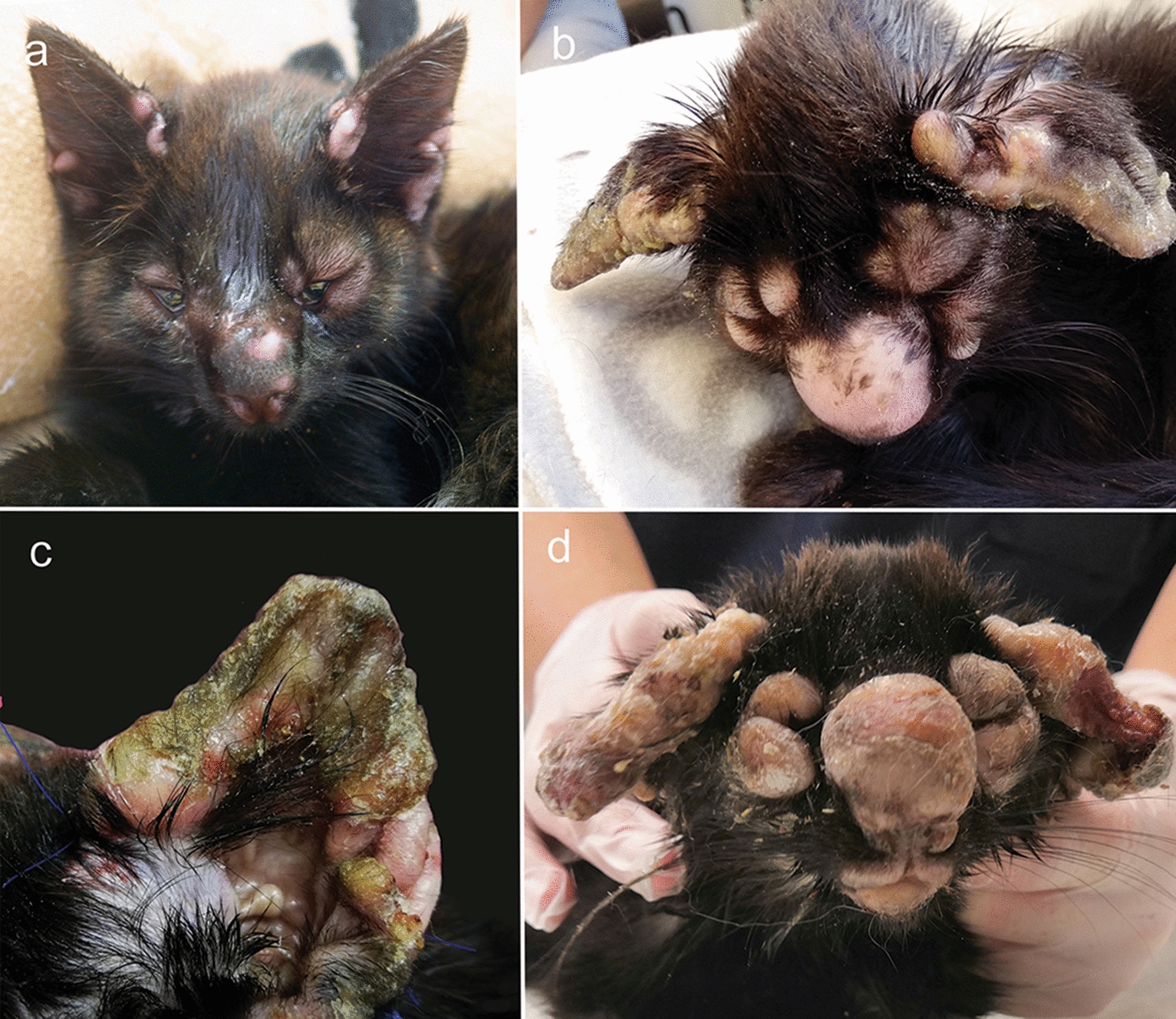
*Caryospora bigenetiga* infection in a cat. The rapid progression of the disease from multiple small nodules to marked firm alopecic swelling of the bridge of the nose, cheeks, and eyelids is shown. The edges of the pinnae were severely thickened with a confluent yellow crust on the concave aspects. **a** Was taken at the time of adoption, **b** and **c** at the time of referral, and **d** at euthanasia

A stray kitten, its dam, and sibling were acquired by a cat shelter. The kitten had a few alopecic nodules at the base of one ear and on the nasal bridge (Fig. 1a). Facial dermatitis progressed quickly after adoption, and the kitten was subjected to veterinary examination. Apart from the dermatological findings, clinical examination revealed no abnormalities. Blood leukocyte count was normal. Tests for anti-feline immunodeficiency virus (FIV) antibodies, feline leukemia virus (FeLV) antigen, and qPCR from a conjunctival swab for *Chlamydophila felis*, feline calicivirus, feline herpesvirus, and *Mycoplasma felis* were all negative. Abdominal ultrasound was unremarkable on a follow-up visit. Total thyroxin was within reference ranges, no growth occurred on a bacterial culture taken by swab from a skin lesion (sample site unknown, sample examined at a referral laboratory), and total thyroxin was within reference ranges. The initial treatment consisted of an injection of methylprednisolone acetate and a course of amoxicillin/clavulanate (dose unknown), but the condition worsened. Treatment was changed to doxycycline (6.2 mg/kg PO q12h), which led to slight temporary improvement, i.e., less swollen eyelids, and pinnae.

The kitten was referred to a veterinary dermatologist 10 days after the initial veterinary examination due to the progression of the skin lesions. There, on general examination, the kitten was thin but alert. On dermatological examination, marked, firm, alopecic swelling of the bridge of the nose, cheeks, and eyelids was present, obscuring the eyes and distorting normal anatomy (Fig. 1b). The edges of the pinnae were severely thickened with firm alopecic nodules, and on the concave aspects, a confluent yellow crust was present (Fig. 1c). Facial lesions were strikingly symmetrical. Multifocal alopecic nodules were present on the limbs, and the distal third of the tail was swollen and alopecic. Two wedge biopsies and cytological and bacteriological samples were taken from the pinnal lesions.

**Fig. 2:**
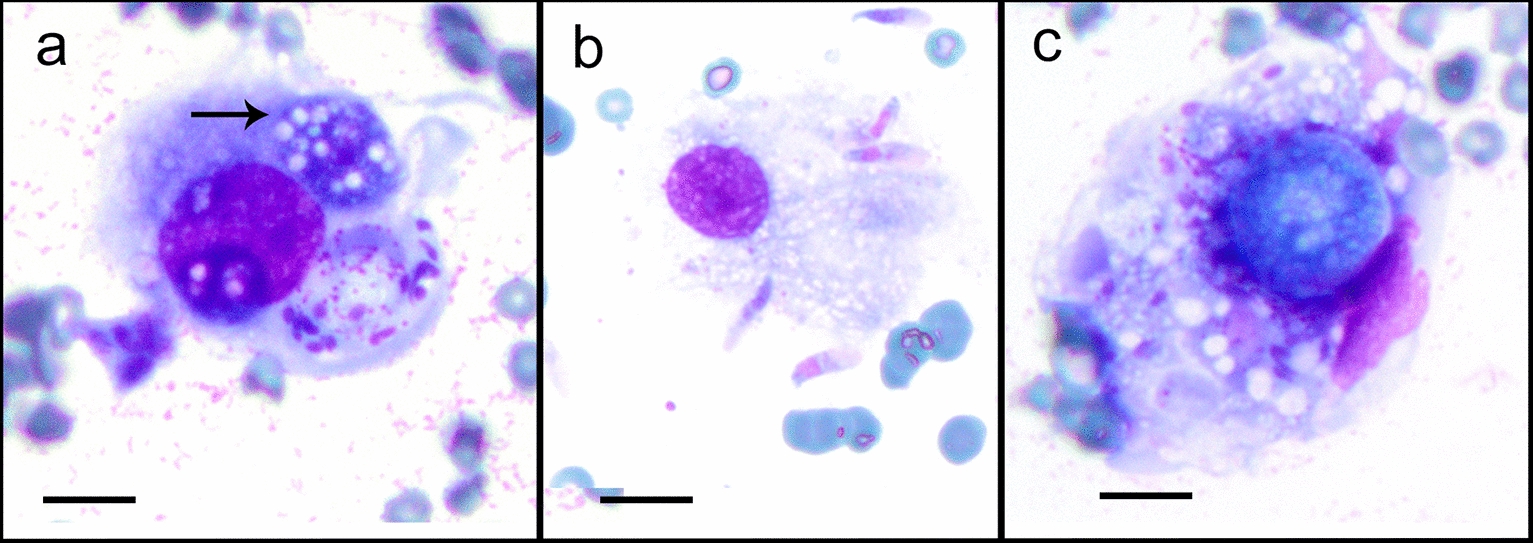
Cytology of the cutaneous *Caryospora bigenetica* infection, May Grünwald-Giemsa stain, scale bar = 10 µm. **a** A caryocyst (arrow) within a macrophage consisting of a large, nucleated sporozoite with an abundant, intensely basophilic cytoplasm with vacuoles. **b** A macrophage with numerous spindle-shaped tachyzoites. **c** A macrophage with an unsporulated oocyst, a capsulated cluster of foamy spherules with a cyan-blue hue

Cytology (Fig. 2a–c) showed pyogranulomatous inflammation with macrophage dominance. Macrophages were filled with numerous pleomorphic organisms with morphology varying from tiny ovoid basophilic structures to large foamy spherules and distinct banana-shaped tachyzoites (tachyzoic merozoites).

**Fig. 3:**
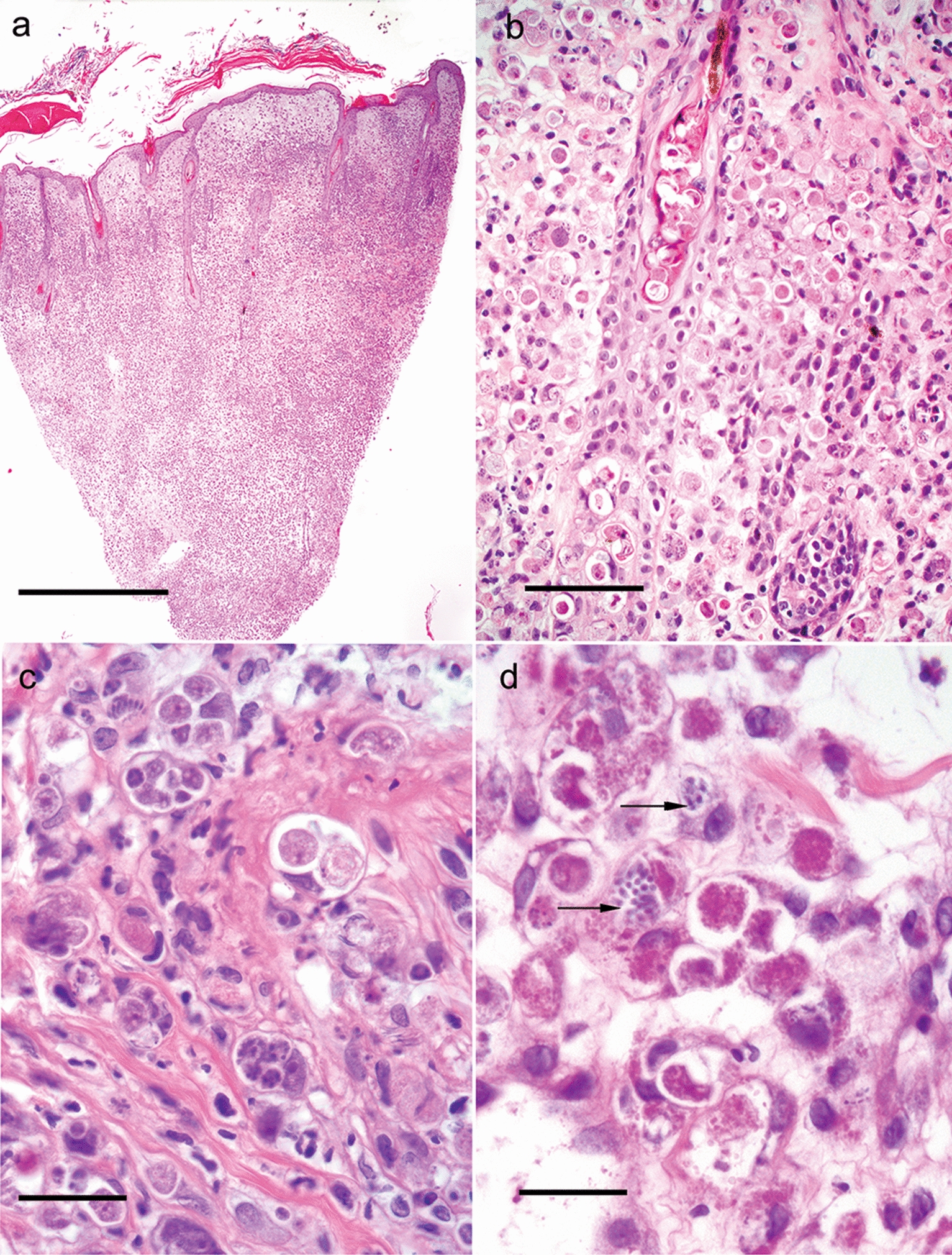
Histopathology of the skin in cutaneous *Caryospora bigenetica* infection. **a** A punch biopsy showing a highly cellular, transdermal, severe diffuse interstitial inflammatory infiltrate effacing the architecture, scale bar = 1 mm. **b** Different developmental stages of *C. bigenetica* are numerous throughout the dermis in macrophages and present within the keratinocytes of hair follicles, scale bar = 50 µm. **c** A detail of the dermal histiocytic inflammatory infiltrates effacing the dermal architecture. Protozoal parasites are present and reproducing within all macrophages, scale bar = 20 µm. **d** Periodic Acid Schiff (PAS) stain highlights *Caryospora* organisms, except for the zoites (arrows). The most abundant stage is an unsporulated oocyst presented as a capsulated aggregate of intensively staining material, scale bar = 20 µm

On histopathology (Fig. 3a–c), the epidermis was edematous and ulcerated. A highly cellular, transdermal, severe diffuse interstitial inflammatory infiltrate was present, effacing the normal architecture of the skin. The inflammatory reaction consisted mainly of macrophages with pale and granular cytoplasm. Basophilic protozoal organisms representing developmental stages of an apicomplexan protozoan life cycle were abundant within macrophages and present within the basal and spinous layers of the epidermis and follicular epithelium, in fibroblasts, and endothelial cells. A few organisms were also present extracellularly. The most abundant stage was an unsporulated oocyst presenting as a capsulated aggregate of foamy, light basophilic, or light purple material, but all stages of the asexual and sexual division of apicomplexan protozoa could be identified. Periodic acid Schiff (PAS) stain highlighted the organisms, except for the zoites (Fig. 3d).

The bacterial culture yielded *Acinetobacter nosocomialis*, identified through MALDI-TOF mass spectrometry (Laboratory of Clinical Microbiology of the Faculty of Veterinary Medicine, University of Helsinki).

Due to suspicion of a *Toxoplasma gondii*-like infection, treatment was switched to clindamycin (13.3 mg/kg PO q12h). The shelter reported that the kitten was playful and maintained its appetite. *Toxoplasma gondii* antibody titer measurements gave a borderline result of 1:32 for IgG, while IgM antibodies were negative.

However, the kitten then lost its appetite, and the skin condition worsened, with the cat being euthanized 2 weeks after referral. At the time of euthanasia, the swollen eyelids obliterated the eyes (Fig. 1d). Both ears were severely thickened, swollen, and heavily bending forward. Supplementary biopsy samples for histopathology, transmission electron microscopy (TEM), and PCR were obtained at euthanasia. A complete necropsy was not performed, but the owner gave consent for taking postmortem tissue samples by the attending veterinarian. Histopathological samples obtained from the skin, oral mucosa, and internal organs (submandibular lymph nodes, popliteal lymph nodes, liver, kidney, spleen, lung, and heart) after euthanasia indicated that the infection had been confined to the skin and the gingival mucosa.

**Fig. 4:**
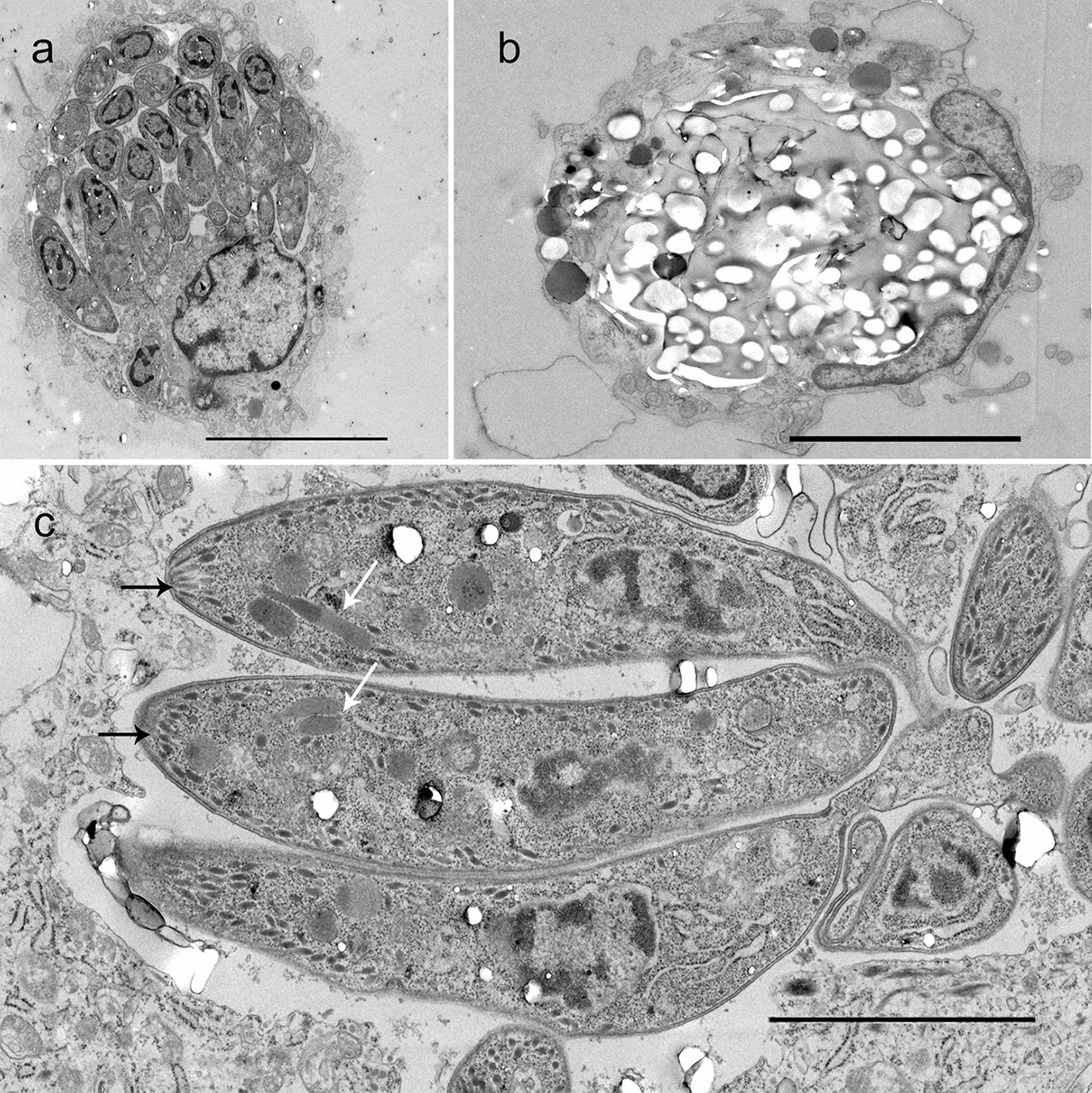
Ultrastructure (transmission electron microscopy) of cutaneous *Caryospora bigenetica* infection. **a** A macrophage occupied by a meront with immature merozoites, scale bar = 5 µm. **b** An unsporulated oocyst within the macrophage consisting mainly of amylopectin granules and lipids, scale bar = 5 µm. **c** Three *C. bigenetica* tachyzoites (tachyzoic merozoites) in the cytoplasm of a macrophage. Banana-shaped appearance, supra-basilar nucleus, and the apical part with a conoid ring, radially orientated rod-like micronemes (black arrows), and obliquely orientated rhoptries (white arrows) are typical morphological features of coccidian apicomplexan protozoa, scale bar = 2 µm

Ultrastructurally, the developmental stages of the parasite appeared typical of apicomplexan protozoa (Fig. 4).

Total DNA was extracted from an alcohol-fixed tissue sample, and a nested PCR amplifying 18S rRNA locus was performed using a nested PCR protocol described by Yang et al. [6]. After the purification of PCR products, sequencing was performed by the Sequencing Unit, Institute for Molecular Medicine Finland FIMM Technology Centre, University of Helsinki. The results of the sequencing reactions were analyzed and edited using Snap Gene Viewer (SnapGene software from Insightful Science; available at https://www.snapgene.com/snapgene-viewer, date of use 9 September 2020). Sequences were compared with existing apicomplexan parasite 18S sequences in GenBank using BLAST searches. Sequence analysis of the 500 base pair amplicon showed 501/501 (100%) homology with *C. bigenetica* (GenBank accession number KT184332.1). Hits with a lower percentage of homology were recorded with *Isospora wiegmanniana* and *Schellackia orientalis* 18S ribosomal gene partial sequences.

## Discussion and conclusions

Reports of dermatitis caused by apicomplexan protozoa with intralesional parasites are rare in the cat. The best-known entity is dermatitis associated with *T. gondii*. In a case series consisting of 100 histologically confirmed feline clinical cases of toxoplasmosis, two cases out of 100 involved skin lesions, but *T. gondii* were not present intralesionally [7]. One disseminated toxoplasmosis case was reported with multiple cutaneous nodules [8], a second case with a single nodule with necrotizing inflammation affecting the skin and mammary tissue [9], and the third case with cutaneous ulcers and hyperemic nodules [10]. In all cases, intralesional *T. gondii* organisms were detected. As an experimental infection has shown that *Neospora caninum* can cause a generalized disease in the cat [11], *N. caninum* is invariably referred to as a possible causative agent when tachyzoites are found in inflamed feline skin. The possibility of *N. caninum* must be considered, as most case reports note positivity, and hence, cross-reactivity, for both anti-*T. gondii* and anti-*N. caninum* antibodies in immunohistochemistry and serology. In addition, one feline case has been described with a disseminated protozoal infection with nodular skin lesions and intralesional tachyzoites. As the organisms exhibited morphologic characteristics of both *N. caninum* and *T. gondii*, they were referred to as *T. gondii*-like organisms [12]. These cases resemble the clinical presentation, histological, cytological, and TEM findings of the present case.

**Fig. 5:**
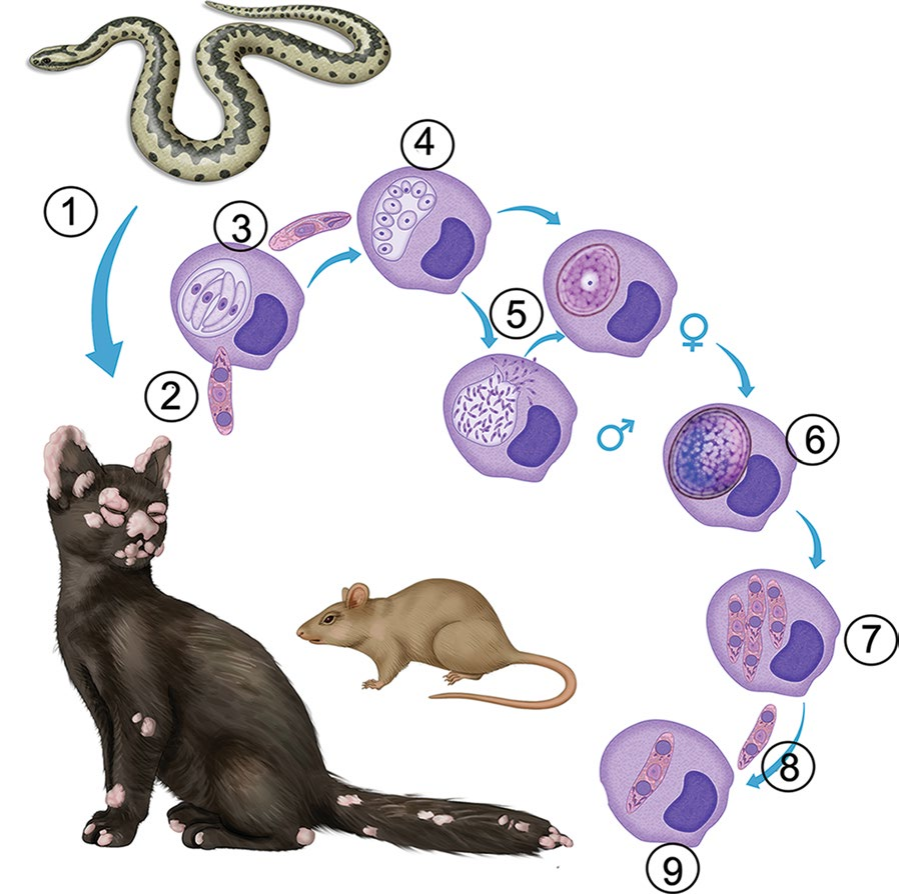
Life cycle of *Caryospora bigenetica*. The parasite infects the intestinal epithelium of snakes, where asexual (merogony) and sexual (gametogony and oocyst formation) reproduction take place. (1) Unsporulated oocysts are shed in the feces of the definitive reptilian host and become infective through sporulation. Ingestion of the sporulated oocysts by the definitive host (snake-to-snake infection) results in intestinal caryosporosis with asexual and sexual multiplications. However, heteroxenous predator–prey cycle (snake-to-mouse-to-snake) may play a vital role in the life cycle in nature. Ingestion of sporulated oocysts by the prey results in extraintestinal asexual and sexual multiplication through merogony, gametogony, fertilization, sporulation, and formation of caryocysts. In the secondary or intermediate host, parasitic stages are found mainly in the skin and mucocutaneous epithelium. In the present case, the kitten played a role as a secondary host. In histopathology, all stages of *C. bigenetica* described in experimental infections could be identified. (2) Sporozoite entering a macrophage, (3) type 1 meront, (4**)** type 2 meront with tachyzoites (tachyzoic merozoites), (5) gamonts, (6) unsporulated oocyst, (7) sporulated oocyst, (8) sporozoite, and (9) caryocyst. Caryocysts and the abundance of unsporulated oocysts were specific findings in this case. The heteroxenous life cycle is completed when a snake ingests an infected secondary host. As the secondary hosts may get the infection not only from oocysts voided by the snake (primary host) but also by consuming other secondary hosts bearing caryocysts, the kitten may have gained the infection feco-orally (snake-to-kitten route) or by ingesting a secondary host (snake-to-mouse-to-kitten route). Also, an unlikely transplacental and galactogenic infection from the queen that got the infection and transmitted the infection to the kitten must be considered (Portions of the life cycle illustration have been modified from Gardiner et al. [2])

The parasites of the genus *Caryospora* are coccidian intracellular intestinal parasites among reptiles and raptorial birds. *Caryospora* may utilize primary hosts' prey animals as secondary hosts [1, 2]. The source of the infection in the present case remains unknown. *Caryspora bigenetica* infection has not been previously documented in Finland. Only two snake species occur in Finnish mainland wildlife: a viper (*Vipera berus*) and a grass snake (*Natrix natrix*). They are common and widespread in Finland, but their intestinal parasites have not been studied. However, several Viperidae species may serve as hosts for *Caryospora* spp. [1]. Experimental infections have shown that the secondary hosts may acquire the infection from oocysts voided by the snake (primary host) or by consuming infected secondary hosts bearing caryocysts [1, 3]. Hence, the kitten may have gained the infection feco-orally (snake-to-kitten route) or by ingesting a secondary host (snake-to-mouse-to-kitten route). Also, an unlikely transplacental and galactogenic infection from the queen transmitted to the kitten (snake/mouse-to-dam-to-kitten route) is possible, although the queen and the sibling showed no signs of infection. Figure 5 illustrates the life cycle stages occurring in the secondary host.

If the parasite’s mouse-to-snake cycle played an essential role in nature and secondary hosts could get infected by consuming an infected mouse, we should see feline cases more often, as is the case with feline *T. gondii*, *Toxocara cati*, and *Isospora* spp. infections. The lack of earlier case reports suggests that *C. bigenetica* is presumably an opportunistic pathogen requiring an immunocompromised cat to manifest infection.

The disease progression from single nodular skin lesions to severely debilitating and deforming skin disease was rapid. Young age may predispose to *C. bigenetica* infection, as seen in dogs [4, 5]. Injecting a long-acting corticosteroid after disease onset may have affected the clinical course, as has been shown in cats experimentally infected with *N. caninum* [10]. However, it is unlikely that the age or the stress of being a stray cat caused significant immunosuppression to result in such a rare and severe disease; hence, an unnoticed immunodeficiency was suspected.

Clinical differential diagnoses considered were a deep bacterial, fungal, or protozoal infection. The bacterial culture yielded *A. nosocomialis*. Fatal infection with *Acinetobacter baumannii* has been reported in a cat [13]. In the present case, *A. nosocomialis* was considered a cause of secondary infection.

The skin lesions of the feline case described here resembled the *C. bigenetica-*associated lesions reported in other species. Infection with *C. bigenetica* in a puppy resulted in multifocal non-alopecic papules and nodules on the head, trunk, and limbs. The pinnae, periorbital region, and muzzle were affected [4]. In an experimental study, mice inoculated with oocysts exhibited swollen muzzles, footpads, scrota (males), partially closed eyelids, and lethargy 8 days after infection [3]. In the same study, infected pigs displayed erythematous eyelids, followed by cutaneous erythema and edema, and all pigs appeared lethargic [3].

The definitive species identification required sequencing of the PCR product. At the 18 S locus, the present isolate exhibited 100% homology without gaps with the *Caryospora cf. bigenetica* strain, and the molecular diagnosis was considered certain.

Had PCR and sequencing been unavailable, the abundance of unsporulated oocysts and caryocysts and the parasite's effective reproduction within macrophages and several other cell types comprised findings that might have allowed differentiation from other protozoal infections and a presumptive diagnosis based on cytology or histopathology. In the histological sections studied, almost all macrophages contained protozoa. Detecting mature tachyzoites enabled narrowing the differential diagnoses to apicomplexan protozoa, *T. gondii*, and *N. caninum* as the most likely protozoa. However, the zoites were sparse in the present case, the smaller immature merozoites in macrophages resembled *Leishmania*’s amastigotes, and unsporulated oocysts resembled *Coccidioides*’ spherules; hence, several infectious differential diagnoses had to be considered.

On TEM, the ultrastructural morphology of the zoites was typical of apicomplexan coccidian protozoa, but TEM did not allow species- or genus-level identification. The number of rhoptries in the tachyzoites varied but finding parasites primarily within membrane-bound vacuoles in the host cells was an ultrastructural feature resembling more *T. gondii* than *N. caninum* [14].

Therapy with a moderately high dose of clindamycin was initiated due to suspicion of *Toxoplasma gondii*-like protozoal infection. A young Rottweiler with cutaneous caryosporosis responded to clindamycin 20 mg/kg and trimethoprim-sulfamethoxazole 23 mg/kg PO q12h [5]. The disease relapsed after treatment was discontinued. Due to the rapid progression and delay in identifying the organism, further treatment modalities were not commenced in this case.

The case described within adds one more apicomplexan parasite to the list of causative agents of protozoal dermatitis in cats and shows that the cat can serve as a secondary host for *C. bigenetica*.

## Data Availability

The data sets used and/or analyzed during the study are available from the corresponding author upon reasonable request.
